# Comparison of Random Periareolar Fine Needle Aspirate versus Ductal Lavage for Risk Assessment and Prevention of Breast Cancer

**DOI:** 10.1007/s12609-012-0081-9

**Published:** 2012-06-22

**Authors:** Abigail Hoffman, Rod Pellenberg, Catherine Ibarra Drendall, Victoria Seewaldt

**Affiliations:** 1 Duke University Medical Center, Box 2628, Durham, NC 27710 USA; 2 Duke University Medical Center, Box 3090, Durham, NC 27710 USA

**Keywords:** Ductal lavage, DL, Random periareolar fine needle aspiration, RPFNA, Breast cancer risk, Breast cancer prevention, Biomarkers, Mammary atypia

## Abstract

Random periareolar fine needle aspiration (RPFNA) and ductal lavage (DL) are research techniques developed to (1) assess short-term breast cancer risk in asymptomatic women who are at increased risk for breast cancer and (2) track cytological response to risk reduction strategies. RPFNA and DL provide minimally invasive methods to repeatedly sample epithelial cells and research tools to investigate the biological origins of breast cancer in high-risk women. This review gives an overview of the strengths and limitations of both RPFNA and DL for risk assessment and breast cancer prevention in asymptomatic high-risk women.

## Introduction

A woman’s breast cancer risk increases with a first-degree family history of breast cancer, a Gail risk score of 1.66 or greater, *BRCA1/2* genetic mutation, or the presence of atypical hyperplasia. There is also evidence that cytological atypia in breast fluid or breast aspirate increases a woman’s risk of subsequently developing breast cancer. Random periareolar fine needle aspiration (RPFNA) and ductal lavage (DL) are minimally invasive research tools that are currently being utilized in a variety of clinical trials to test for the presence of cytological atypia in high-risk asymptomatic women and to track response to risk reduction strategies.

Breast cancer incidence has been shown to be reduced in high-risk cohorts by chemoprevention agents such as tamoxifen and through prophylactic surgery [1–4]. However, not all risk reduction strategies are effective in all women, and moreover, they may carry potential side effects. Furthermore, our current clinical trial design makes it difficult to prospectively identify individual women who are responding to a risk reduction intervention or a prevention agent. The length of time required for prospective validation of a predictive biomarker is not an efficient method for implementing safe and effective therapeutic treatments. Emerging evidence suggests that combined interventions such as weight loss, exercise, and a targeted prevention agent may be more effective than a single intervention alone. As a result, there is an increasing need to identify biomarkers that will accurately predict short-term breast cancer risk in individual women and rapidly assess response to complex risk reduction strategies.

Biomarkers that vary with risk and response to prevention interventions are referred to as *surrogate endpoint biomarkers* [5]. As has been outlined by Fabian et al. [6], surrogate endpoint biomarkers should be (1) biologically and statistically significantly associated with cancer development, (2) present in a reasonable proportion of at-risk individuals, (3) obtainable by minimally invasive procedures, and (4) reversible with prevention interventions that have been validated to decrease cancer incidence. Many modalities have been suggested as potential surrogate endpoint biomarkers for breast cancer, including mammographic density, serum biomarkers, and breast tissue biomarkers [7–10]. Currently, there is no consensus as to the optimal surrogate endpoint biomarker.

Breast tissue biomarkers offer the advantage of directly testing for precancerous changes in the breast. Atypia and lobular carcinoma in situ (LCIS) are associated with increased breast cancer risk [11]. Moreover, breast cancer incidence in women with atypical hyperplasia or LCIS is substantially reduced after treatment with tamoxifen [1, 2]. However, the optimal method to repeatedly sample breast tissue remains controversial. Repeated random core needle biopsies for risk surveillance and/or for measurement of response to a prevention intervention can cause significant patient discomfort and are problematic because, unless the biopsy specimens are obtained from mammographically dense areas, the biopsy is likely to contain few terminal ductal–lobule units [12]. Nipple aspirates have shown some promise. However, approximately 40 % of nipple aspirates are acellular [13]. Here, we aim to review the strengths and limitations of two research techniques, RPFNA and DL, that have been developed to repeatedly sample mammary epithelial cells and to test surrogate biomarkers of response to prevention in individual high-risk women.

## Random Periareolar Fine Needle Aspiration (RPFNA)

RPFNA is a research technique that was developed by Carol Fabian, M.D., at the University of Kansas in the mid-1980s to (1) assess short-term breast cancer risk in women at high risk for breast cancer and (2) track cytological response to risk reduction strategies [6, 14]. RPFNA is distinct from diagnostic FNA. Whereas diagnostic FNA is a standard clinical technique used to evaluate a clinically identifiable breast mass, breast RPFNA aims to provide a sampling of cells from the entire breast of asymptomatic women. Therefore, RPFNA has the advantage of being able to provide a “snap-shot” of the whole breast. The strengths of RPFNA are that (1) the technique can be performed successfully in a majority of high-risk women (72 %–85 % cell yield) and (2) the presence of cytological atypia in RPFNA has been shown to prospectively predict short-term breast cancer risk in high-risk women [15–17].

In 1986, the late Helene Smith proposed that breast cancer developed in a “high-risk field” or segment of the breast containing molecular changes that promote the development of a malignancy [18]. The existence of a “high-risk field” remains controversial; however, it is clear that when proliferative changes are present in the breast, these changes occur in a multifocal and multicentric pattern [19–21]. RPFNA has the ability to sample multifocal proliferative changes and to evaluate potential “field-effects.” This is particularly useful for short-term prevention trials because the aim of these studies is not to biopsy or treat a focal lesion but, rather, to induce antiproliferative or biomarker changes throughout an at-risk breast or within a “high-risk field.”

### Performance of RPFNA

**Fig. 1:**
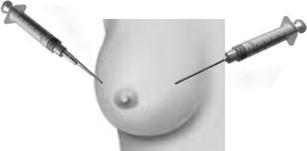
Random periareolar fine needle aspiration. The breast is anesthetized with 5 cc of 1 % lidocaine, 2–5 cm from the areola (depending on the size and shape of the breast), at approximately three and nine o’clock. Nine aspirations are performed per breast, with four aspirations from the medial skin site and five from the lateral breast site

The breast is anesthetized with 5 cc of 1 % lidocaine, 2–5 cm from the areola (depending on the size and shape of the breast), at approximately three and nine o’clock. Nine aspirations are performed per breast, with four aspirations from the medial skin site and five from the lateral breast site. While aspirations are guided by the tactile identification of stromal/ductal tissue, there is no attempt to aspirate a specific location within the breast (Fig. 1). Aspirated cells contain a mixture of epithelial, stromal, adipose, and immune cells. These cells are pooled and placed in modified CytoLyt^TM^ with 1 % formalin for 24 h. Carol Fabian’s group pools cells from both breasts into a single sample [6], whereas, the Cancer and Leukemia Group B (CALGB) Prevention Group pools cells from the right and left breast separately, so as to obtain one sample per aspirated breast [22•].

### Masood Score Evaluation of RPFNA Cytology

RPFNA epithelial cytology is most frequently evaluated on thin layer cytologic preparation using the Masood Cytology Index score, a semiquantitative, numeric assessment scale that is weighted toward epithelial nuclear morphology. A minimum of one epithelial cell cluster with >10 epithelial cells is required for assessment, and the most abnormal cell cluster is scored [16, 17]. Epithelial cells are given a score of 1 to 4 points for each of the six morphological characteristics: cell arrangement, pleomorphism, number of myoepithelial cells, anisonucleosis, nucleoli, and chromatin clumping. The sum of these points computes the Masood score: <10, nonproliferative (normal); 11–13, hyperplasia; 14–17, atypia; and >17, suspicious cytology [16, 17]. The numbers of epithelial cells are quantified and classified as <10 cells (insufficient), 10–100 cells, 101–500 cells, 501–1,000 cells, 1,001–5,000 cells, and >5,000 cells. The number of epithelial cells increases with the degree of cytological atypia; epithelial cell yield ranges from (1) 10^4^ to 5 × 10^6^ (Masood 11–13), (2) 10^6^ to 5 × 10^8^ (Masood 14–17), and (3) 10^7^ to 10^9^ (Masood >17). Accordingly, epithelial cytology from repeat samples possibly provides the ability to examine risk biomarkers and to prospectively predict the subsequent development of breast cancer.

### Predictive Ability of RPFNA to Prospectively Identify Short Term Breast Cancer Risk

In 2000, Carol Fabian conducted a single institution study that demonstrated that cytological atypia in RPFNA independently predicted for an increase in breast cancer risk in high-risk women [6]. In this trial, 480 women at increased risk for breast cancer on the basis of (1) family history of breast cancer, (2) a prior diagnosis of breast cancer, or (3) precancerous biopsy prospectively underwent RPFNA. Investigators tested for the ability of cytological atypia in the initial RPFNA aspiration and Gail risk score to prospectively predict the subsequent development of breast cancer. At a 45 months follow-up, 20 women developed breast cancer. Of these, there were 7 DCIS and 13 invasive breast cancers. Multiple logistic regression and Cox proportional hazards analysis demonstrated that either the presence of cytological atypia in the initial RPFNA or a 10-year Gail projected an increased probability of developing breast cancer. The presence of cytological atypia in RPFNA predicted for a 5.6-fold increase in the subsequent development of breast cancer. This study supported the use of cytological atypia in RPFNA as a marker for high-risk benign lesions in high-risk women and supported the use of RPFNA in subsequent risk assessment and prevention studies [6].

### Advantages and Limitations of RPFNA

RPFNA has the advantages of being inexpensive, performed in a clinic exam room, and repeatable, allowing for the serial assessment of predictive biomarkers. The majority of RPFNA aspirates in high-risk premenopausal and perimenopausal women are cellular [23, 24]. As was described above, the study of Fabian et al. prospectively validated the use of cytological atypia in RPFNA as a surrogate risk marker in high-risk women. However, the study of Fabian et al. has limitations, because it is single-institution study and cannot be generalized to average-risk women. No study has been performed to independently confirm the ability of cytological atypia in RPFNA to predict development of breast cancer.

While RPFNA has gained in acceptance, its use is limited by the absence of data with respect to reproducibility in the technique and interpretation in multiinstitutional studies. To address these potential limitations, the CALGB Prevention Group tested the reproducibility of RPFNA in a multiinstitutional cross-sectional study of high-risk women [22•]. In this study, Masood Cytology Index scores and two cell count measurements were compared from RPFNA samples of 63 individual women from five institutions (Duke University, Ohio State University, Roswell Park, University of Vermont, and Dana Farber Cancer Institute). All investigators were individually trained to do RPFNA by Carol Fabian at the University of Kansas. RPFNA was performed on the same breast, on the same day, by the same investigator, using separate needles for sequential aspirations. Masood Cytology Index score and epithelial cell count were assigned by a blinded, single dedicated cytopathologist (C.M.Z.). In this study, RPFNA was found to be highly reproducible with the overall Spearman correlation coefficients for Masood Cytology score and cell count of .8312 (*p* < .0001) and .7260 (*p* < .0001), respectively. Importantly, the reproducibility of duplicate RPFNA samples from the same breast was not affected by age, body mass index, 5-year Gail risk score, menopausal status, *BRCA1/2* mutation, or the number of first-degree family members with breast and/or ovarian cancer [22•].

Studies performed by the CALGB Prevention Group provide evidence that RPFNA measurements are reproducible in a cooperative group setting and support the future use of RPFNA in multiinstitutional prevention trials. However, there were several limitations to this study. First, the number of subjects was small (63 women). Second, intraoperator variability of RPFNA was assessed, but the interoperator variability was not. The concordance between RPFNA measurements provided evidence that single operators in multiple institutions can produce similar results. But this study did not address whether different operators would have similar findings in the same woman. Despite the limitations of this study, these data provide important validation of the reproducibility of RPFNA in a multiinstitutional cross-sectional study that included cohorts that varied in demographic composition [22•].

### Biomarker and Prevention Studies Utilizing RPFNA

Carol Fabian’s group pioneered the use of RPFNA-based biomarkers [14]. The first prevention study utilizing RPFNA-based biomarkers was a double-blind randomized Phase II chemoprevention trial [14] of alpha-difluoromethylornithine (DFMO) in 119 women at high risk for the development of breast cancer. Prior to entry, women were required to have RPFNA cytology that exhibited hyperplasia or hyperplasia with atypia, as well as a mammogram and clinical breast exam judged as not suspicious for breast cancer. Subjects were randomized to take 0.5 g/m^2^ DFMO or placebo p.o. once per day for 6 months, followed by a repeat RPFNA. Of 119 subjects entered, 96 % completed the study and were evaluable for the main study end point [14]. The trial demonstrated no change in RPFNA cytology and RPFNA-based secondary end points of breast molecular marker changes (immunocytochemical [IC] expression of proliferating cell nuclear antigen, p53, and epidermal growth factor receptor) [14]. Since then, numerous studies have tested expression of IC and methylation biomarkers in RPFNA epithelial cytology [14, 19–21, 22•, 23–30]. The key limitation of all RPFNA-based biomarkers is the heterogeneous nature of the cell populations being tested for specific molecular markers. Recently, investigators have performed investigator-blinded proteomic profiling of RPFNA epithelial cytology, using the reverse phase proteomic microarray (RPPM) [31•, 32•]. Up to 60 phosphoproteins can be tested in triplicate from 5,000 to 10,000 microdissected RPFNA epithelial cells. These studies provide the feasibility for tracking phosphoprotein network signaling in RPFNA cytology in response to risk reduction strategies, as well as having the potential to increase our understanding of the earliest events in mammary carcinogenesis in high-risk women. For example, the RPPM analysis of epithelial cells from RPFNA samples of nonobese and obese high-risk women has identified a potential cross-talk between the receptor tyrosine kinase/mammalian target of rapamycin signaling with interleukin-6/signal transducer and activator of transcription signaling in vimentin expression (unpublished results). Given the established associations between obesity and adipokines in breast cancer risk, future proteomic studies will employ serum and breast RPFNA samples to determine protein expression changes in the aforementioned signaling networks of high-risk women undergoing preventative interventions, such as diet and exercise. While this strategy appears promising, large-scale adoption of RPPM-RPFNA-based biomarkers will depend on robustness and reproducibility in investigator blinded, multiinstitutional trials.

## Ductal Lavage (DL)

DL is an experimental technology that utilizes nipple fluid to identify high-risk and occult malignant lesions in breast epithelial cells. Initial data from a multicenter study by Dooley et al. [33] established the safety and tolerability of the procedure and increased cell yield for diagnostic evaluation, as compared with nipple aspirates. Five hundred seven high-risk women were enrolled, including 57 % with a history of contralateral breast cancer and 39 % with a 5-year Gail risk of >1.7 %. NAF production was possible in 83 % of these women, and 77 % of these women had DL performed, yielding adequate evaluable epithelial cells for cytologic diagnosis in 60 % (299/507) of the patients. The median number of epithelial cells from evaluable NAF specimens was 120 cells per breast, as compared with 13,500 epithelial cells per duct with DL. DL was 3.5 times more successful at producing cytologically evaluable fluid, as compared with NAF (73 % vs. 22 %, respectively; *p* < .001), and therefore, offered a more sensitive method of detecting cellular atypia [33]. Cytology findings for DL demonstrated inadequate cellular material for diagnosis in 22 %, benign in 54 %, mildly atypical in 17 %, and markedly atypical or malignant in 7 % of patients. The cytopathology results are intended for detection of epithelial atypia to improve the precision of breast cancer risk assessment, for detection of occult malignant lesions, and for potential serial observation in chemoprevention clinical trials.

### Performance of Ductal Lavage

**Fig. 2:**
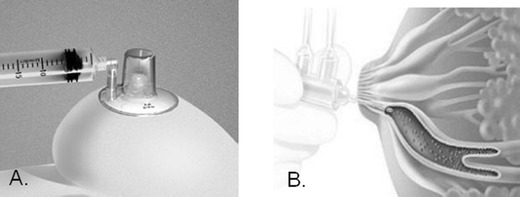
Ductal lavage. **a** Nipple fluid aspirator. The procedure begins with breast massage and placement of a suction device to the nipple to identify fluid-yielding ducts and visualize non-fluid-yielding ducts. **b** Single lumen duct lavage connected to inflow and outflow syringes. Saline is infused through the inflow syringe and then aspirated to collect cells from the lining of the milk duct

The procedure begins with breast massage and placement of a suction device to the nipple to identify fluid-yielding ducts and visualize non-fluid-yielding ducts, which are then cannulated with a microcatheter, infused with saline or physiologic solution, and then aspirated to collect cells from the lining of the milk duct (Fig. 2). The location of the lavaged duct is recorded on a grid to mark the position for future, repeat lavage. The resultant fluid is then analyzed for cytopathology and categorized as insufficient cellular material for cytologic diagnosis, benign, mild atypia, severe atypia, or malignant.

### Predictive Ability of Ductal Lavage to Prospectively Identify Short-Term Breast Cancer Risk

The potential for early detection of breast cancer by DL was examined in 2004 by Khan et al. [34] in 39 women with known breast cancer undergoing mastectomy. In this study, lavage was possible in 36 (82 %) of 44 breasts, and 31 (70 %) specimens had sufficient cellularity, excluding specimens that were acellular or contained fewer than 100 epithelial cells. A high-threshold positive test, defined as markedly atypical or malignant cytology, was found in only 13 % (5/38) of cancerous breasts, whereas a low-threshold positive test that additionally included mildly atypical cytology was present in 42 % (16/38) of cancerous breasts. Therefore, the sensitivity for cancer detection was 13 %–42 % when marked versus mild atypia was included, which was attributed to cancer-containing ducts failing to yield fluid or having benign or mildly atypical cytology. These results challenge the previous hypothesis that the breast ducts yielding fluid on nipple aspiration are the ones most likely to contain malignant disease. Similar findings were shown in a study performed at Stanford [35] in which 38 women underwent the DL procedure with 30 successful attempts that resulted in 7/30 (23.3 %) with atypical lavage cytopathology. Of the ducts that yielded atypia, five of seven were from ducts that were non-fluid-yielding on initial suction aspiration. In addition, only one of seven patients with atypia in DL fluid had a normal breast MRI and mammogram screening of the affected breast; however, six of seven patients with atypia had normal mammograms [35]. Maddux et al. determined that atypia was equally as likely to be found in both fluid-producing and dry ducts (19 % vs. 15 %) [36]. However, Wood et al. found that nipple aspiration fluid was more commonly expressed in cancerous, as compared with unaffected, breasts. Overall, sensitivity for detecting marked atypia or malignant cells in affected breasts was 17 %, which is comparable to the results in other studies [37].

### Advantages and Limitations of Ductal Lavage

DL is a nonsurgical breast epithelial sampling procedure that was developed to enhance the cellularity and reproducibility for detection of epithelial atypia and for serial observation in chemoprevention trials. DL offers the ability to label individual ducts for future recannulation in order to decrease sample variance in prevention trials, in addition to identifying the location of any abnormal cells found by cytopathology. However, the efficacy of DL for the serial monitoring of breast epithelium was tested for reproducibility in a study of 23 women, indicating that fewer than 50 % of women producing atypical DL samples on the first attempt were found to have atypical samples on a repeat DL attempt [38]. The reliability of DL was also tested by Visvanathan et al. [39] and demonstrated a high rate of inadequate cellular material for diagnosis with fair cytologic reproducibility and low participant return rate. Reproducibility was further examined at Northwestern University in 2008, resulting in comparable low reproducibility of cell yield and cytologic findings after a baseline and 6-month repeat DL was performed in 65 high-risk women. Cytologic diagnosis was reproducible only in 43 % of patients, concluding the limited value of DL for serial monitoring in chemoprevention trials [40].

Tolerability of DL has been reported as a possible limitation to its clinical use. The first multiinstitutional study of DL by Dooley et al. [33] reported the procedure to be well tolerated without any documented serious adverse events. Nonetheless, the study states that 51 % reported DL as less comfortable than mammography, and 44 % experienced breast pain. Another study reported that DL was not well tolerated, in part due to increased pain, as compared with mammography and NAF. There was also a low participant return rate for a repeat DL [39]. In 2009, the tolerability of DL was again examined closely among high-risk women and resulted in increased anticipated, in addition to increased experienced, discomfort versus mammogram, MRI, or NAF. Here, 25 % of women refused to repeat DL [41].

### Biomarker and Prevention Studies Utilizing Ductal Lavage

Optimal breast cancer predictive models are combining biologic and clinical markers to enhance the sensitivity of DL and identify characteristics of malignant changes within the breast. For example, DL was utilized for cancer detection in 2002 by King et al. [42] with 39 paired cases of surgically excised breast lesions and DL specimens collected prior to surgery. The study showed a sensitivity of 47 % and a specificity of 79 % in surgical specimens demonstrating abnormal cytology. Results were enhanced by the use of molecular markers consisting of fluorescence in situ hybridization with probes for aneusomy that showed a sensitivity and specificity of 71 % and 89 %, respectively. Another study that increased the sensitivity of cytology was demonstrated by Fackler et al. [43], who utilized quantitative multiplex methylation-specific polymerase chain reaction (QM-PCR) to quantitate cumulative gene promoter hypermethylation in multiple genes that are markers for breast cancer. As compared with cytology, QM-PCR doubled the sensitivity of detection of cancer cells.

To determine the principle that biomarkers of chemoprevention agents can be examined using serial DL, several phase 2 prevention trials were initiated using tamoxifen. Bhandare et al. [44] assessed the utility of DL by measuring biomarkers of tamoxifen that included estrogen receptor α, Ki-67 and cyclooxygenase-2 and reported baseline findings that acknowledged the feasibility of cytologic and biomarker studies in 168 patients. This phase 2 trial included a baseline DL, and the subject chose tamoxifen or observation, followed by a repeat DL 6 months later. The final results of this trial were reported 4 years later with noteworthy limitations: (1) lack of reproducibility of biomarkers in the observation group, (2) only 53 % retention of patients from recruitment to biomarker analysis, and (3) high cost of DL. In conclusion, DL was labeled as an inefficient method of biomarker measurement in high-risk women in this single-institution study [45].

## RPFNA versus DL

Comparison of DL and RPFNA as tissue acquisition methods in early breast cancer prevention trials was tested prospectively at a single institution by evaluating sample adequacy and patient tolerability [46]. In this study, 86 high-risk women (median age of 54.5 years, 75 % postmenopausal) participated in two prospective phase II chemoprevention trials in which both DL and RPFNA were done at baseline. Only 31 % of subjects yielded adequate DL samples for evaluable analysis versus 96 % for RPFNA, concluding that DL is far less practical as a biomarker in chemoprevention trials, as compared with RPFNA. This study also demonstrated that both procedures were highly tolerable, without the development of hematoma, infection, or postprocedure pain requiring analgesic administration [46]. Similar findings were shown in Zalles et al., with 62 % and 96 % of high-risk women having a successful procedure with evaluable cytomorphology in DL and RPFNA, respectively [16]. Both studies demonstrated that RPFNA was more likely to yield evaluable samples, but each method offered similar cytology results when adequate samples were provided [16, 46].

Evaluation of the cost effectiveness with RPFNA and DL in stratifying women for breast cancer preventive interventions was studied in 2004 and demonstrated effective means to motivate women at high risk for breast cancer to take tamoxifen if atypia were found. In this study, RPFNA was found to be both less expensive and more likely to produce an evaluable specimen, when compared with DL [47]. Furthermore, attempted DL has been dependent on the successful production of NAF for multiple studies, but when compared with concordant RPFNA specimens, atypical cells were observed in women with no NAF or an acellular lavage specimen [48]. Therefore, the lack of production of NAF does not exclude cytological atypia, and another means of screening for breast cancer is necessary.

## Conclusions

Currently, we lack agents to prevent estrogen receptor-negative (ER−) breast cancer. A wealth of targeted inhibitors are undergoing clinical testing for the treatment of ER(−) breast cancer. However, in order to effectively test targeted agents for prevention, we first need biomarkers to identify women who have the highest likelihood of response. Strategies to rapidly test for response and resistance are also needed.

The past 20 years have focused on developing single biomarkers. However, it is clear that normal mammary gland homeostasis requires the coordinated regulation of phosphoprotein signaling networks (rather than single proteins). Currently, we lack an understanding of the biology of breast cancer initiation. The combination of RPFNA or DL with high-throughput proteomic profiling holds promise for investigating whether dysregulation of interconnected signaling networks predicts cancer initiation and progression. This approach may also allow for rapid tracking of response to complex risk reduction interventions.

Low-toxicity targeted agents are potentially useful in the prevention of ER(−) breast cancer. However, emerging evidence suggests that some targeted agents, due to their specificity, may have unanticipated mechanisms of resistance [49]. Recently, it was shown that women receiving lapatinib for invasive breast cancer developed resistance via paradoxical activation of quiescent signaling pathways. Suppression of ErbB2-signaling by lapatinib in ER(−) breast cancer resulted in activation of FOXO3a and caveolin-1, leading to activation of ER-signaling [50]. This result was unexpected and highlights the importance of directly testing for protein signaling in mammary cytology, rather than relying on indirect markers of response, during administration of targeted agents. The combination of RPPM with RPFNA or DL in multiple biomarker development and validation may not only serve to explain the underlying pathology of breast cancer subtypes, but also directly test whether risk reduction strategies benefit individual women.

## Abbreviations

RPFNA: Random periareolar fine needle aspiration
DL: Ductal lavage
NAF: Nipple aspirate fluid
DCIS: Ductal carcinoma in situ
LCIS: Lobular carcinoma in situ
